# Motile aeromonads from farmed and wild freshwater fish in northern Italy: an evaluation of antimicrobial activity and multidrug resistance during 2013 and 2016

**DOI:** 10.1186/s13028-020-0504-y

**Published:** 2020-01-23

**Authors:** Laura Borella, Cristian Salogni, Nicoletta Vitale, Federico Scali, Vittorio Maria Moretti, Paolo Pasquali, Giovanni Loris Alborali

**Affiliations:** 1 Istituto Zooprofilattico Sperimentale della Lombardia e dell’Emilia Romagna “Bruno Ubertini”, Via Bianchi 9, 25124 Brescia, Italy; 2 Department of Veterinary Medicine, University of Milan, Via dell’Università 6, 26900 Lodi, Italy; 3 Department of Veterinary Public Health and Food Safety, Istituto Superiore di Sanità, Viale Regina Elena 299, 00161 Rome, Italy

**Keywords:** Aquatic zoonosis, Critically important antimicrobials, Freshwater fish, Motile aeromonads, Multidrug resistance

## Abstract

**Background:**

Antimicrobial resistant bacteria are emerging biological contaminants of the environment. In aquatic ecosystems, they originate mainly from hospitals, livestock manure and private households sewage water, which could contain antimicrobial agents and resistant microorganisms. *Aeromonas* spp. occur ubiquitously in aquatic environments and they cause disease in fish. Motile aeromonads are also associated with human gastrointestinal and wound infections and fish can act as a transmission route of antimicrobial resistance (AMR) aeromonads to humans. The environmental ubiquity, the natural susceptibility to antimicrobials and the zoonotic potential of *Aeromonas* spp. make them optimal candidates for studying the AMR in aquatic ecosystems.

**Results:**

The AMR patterns of 95 motile aeromonads isolated from freshwater fish during 2013 and 2016 were analyzed. All samples from fish came from farms and natural water bodies located in northern Italy, which is an area characterized by high anthropic impact on the environment. The isolates were biochemically identified as *Aeromonas hydrophila*, *Aeromonas sobria* or *Aeromonas caviae* and AMR was determined by the standard disk diffusion method. All isolates were resistant to cloxacillin, spiramycin and tilmicosin. High AMR frequencies (> 95%) were detected for tylosin, penicillin and sulfadiazine. AMR to danofloxacin, enrofloxacin, flumequine, ceftiofur, aminosidine, colistin, doxycycline, gentamicin, marbocyl and florfenicol was observed at low levels (< 10%). No AMR to cefquinome was found. Logistic regression showed several differences in antimicrobial activity between complexes. According to the source of aeromonads, only few differences in AMR between isolates from farmed and wild fish were observed.

**Conclusions:**

Our data revealed an increasing trend of AMR to neomycin and apramycin among *Aeromonas* isolates during the study period, while resistance to erythromycin, tetracycline and thiamphenicol decreased. All isolates were multidrug resistance (MDR), but *A. caviae* showed the highest number of MDR per isolate. In most isolates, various degrees of MDR were detected to macrolides, quinolones, fluoroquinolones, polymyxins and cephalosporins (third and fourth generations), which are listed, by the World Health Organisation, to be among the highest priority and critically important antimicrobials in human medicine. Our findings underlined that freshwater fish can act as potential source of MDR motile aeromonads. Due to their zoonotic potential, this can pose serious threat to human health.

## Background

The environment is increasingly being recognized for the role it might play in the global spread of antimicrobial resistance (AMR). In particular, aquatic ecosystems may provide an ideal setting for the acquisition and spread of AMR because they are constantly exposed to anthropogenic changes such as sewage water from hospitals, private households and livestock manure. Among intensive type of livestock production, fish farms have a direct impact on the aquatic environment because antimicrobials in supplemented feed as therapeutic agents are added into the water. Drug residues typically remain in aquaculture environments and they may also be carried with effluents from production facilities into open waterways.

The genus *Aeromonas* comprises ubiquitous bacteria that occur normally in aquatic environments [1]. Therefore, they are constantly exposed to the action of micropollutants, such as residual antimicrobial compounds that may be present. Depending on their concentration, contaminating antimicrobials can exert a selective pressure and may thus favour the spread of AMR among aquatic bacterial populations [2].

*Aeromonas* spp. are best known as agents of fish diseases, but motile species are now emerging as important opportunistic human pathogens. They have been associated with several food-borne outbreaks and are progressively being isolated from patients with traveller’s diarrhea [1]. The consumption of contaminated raw or cured fish and fish products is considered the main source of gastroenteritis. Most cases of the diseases that are reported are related to aquaculture products or cold-stored ready-to-eat food [3]. Moreover, motile *Aeromonas* spp. can cause skin and soft tissue infections as a result of injuries when handling fish or working in aquaculture and also when keeping fish as pets, for example as aquarium owners [4].

Given their zoonotic potential, the global rise in AMR of motile *Aeromonas* isolates from different sources may pose a serious threat to public health [5–9]. Despite the vast information on the AMR of motile aeromonads isolated from water, sediments and seafood [5–7, 10–12], few studies exist on the AMR patterns of isolates from fish, which are naturally exposed to these environmental microorganisms.

In order to fill this gap, the present work was designed to study the presence and the degree of AMR in motile aeromonads recovered from farmed and wild freshwater fish in northern Italy and to determine whether there are differences in AMR profiles between isolates from farmed and wild fish. The AMR of *Aeromonas* spp. over the 3-year period 2013–2016 was also evaluated. Finally, the presence of multidrug resistance (MDR), defined as the resistant to at least three classes of antimicrobials, was assessed.

## Methods

### Collection of bacterial strains

A total of 95 motile *Aeromonas* spp. isolates were included in this study. All isolates were recovered from internal tissues (liver, kidney, spleen, or brain) of 26 farmed fish and 69 wild fish of different freshwater species, submitted to the Laboratory of Diagnostic and Animal Health sector of the Istituto Zooprofilattico Sperimentale della Lombardia e dell’Emilia Romagna (IZSLER) in Brescia, Italy during the years 2013 and 2016. All fish samples came from farms and natural water bodies located in the Po River Valley (northern Italy), a geographical area characterized by high anthropic impact on the environment due to agricultural, livestock and industrial activities.

Bacterial strains were isolated on Tryptone Soya Agar (Oxoid, Italy) and Blood Agar (Oxoid, Italy) after 48 h of incubation at 22 ± 2 °C in aerobic conditions.

### Phenotypic identification

All *Aeromonas* isolates were identified at the genus level with a panel of tests according to Martin-Carnahan and Joseph [13]. Subsequently*, Aeromonas* isolates were biochemically typed at complex level by using previously published criteria [14]. According the Abbott scheme (Voges–Proskauer test, esculin hydrolysis, L-arabinose fermentation and gas production from glucose), each isolate was assigned to one of three traditionally recognized complex of motile *Aeromonas* spp.: *Aeromonas hydrophila*, *Aeromonas sobria*, and *Aeromonas caviae*.

The following reference strains were used in parallel with test isolates as positive/negative controls for the interpretation of doubtful biochemical reactions: *Escherichia coli* ATCC 25922, *Pseudomonas aeruginosa* ATCC 27853, *Staphylococcus aureus* ATCC 25923, *Streptococcus agalactiae* ATCC 27956, *Klebsiella pneumonia* ATCC 13883, *Citrobacter freundii* ATCC 43864, *Salmonella enterica* subsp. *salamae* (IZSLER 2009), *Salmonella typhimurium* ATCC 14028, *Proteus mirabilis* ATCC 29906, *Listeria monocytogenes* ATCC 13932.

Stock cultures were maintained at − 20 °C in Trypticase Soy Broth medium (Oxoid, Italy) supplemented with glycerol at 20% (vol/vol).

### Antimicrobial resistance

*Aeromonas* isolates were tested for their susceptibility to a panel of 30 antimicrobials by the disk diffusion method on Mueller Hinton Agar (Oxoid, Italy) [15]. Antimicrobials frequently used in animal husbandry and human medicine were included in the study: aminosidine (10 µg), amoxicillin (25 µg), amoxicillin/clavulanic acid (AMC, 30 µg), ampicillin (10 µg), apramycin (15 µg), cephaloridine (30 µg), cefquinome (10 µg), ceftiofur (30 µg), cloxacillin (1 µg), colistin (10 µg), danofloxacin (5 µg), doxycycline (30 µg), enrofloxacin (5 µg), erythromycin (15 µg), florfenicol (30 µg), flumequine (30 µg), gentamicin (10 µg), kanamycin (30 µg), marbofloxacin (5 µg), nalidixic acid (30 µg), neomycin (30 µg), penicillin (10 UI), spiramycin (100 µg), sulfadiazine (300 µg), tetracycline (30 µg), thiamphenicol (30 µg), tiamulin (30 µg), tilmicosin (15 µg), trimethoprim/sulfamethoxazole (SXT, 25 µg) and tylosin (30 µg). All disks were supplied by Oxoid (Italy). Susceptibility tests were performed with the standard protocols (Clinical and Laboratory Standards Institute, CLSI) [16] using unmodified Mueller–Hinton media incubated at 22 ± 2 °C for 24 h, as suggested by Smith [17] for non-fastidious Gram-negative bacteria. The incubation conditions recommended in the standard methods for AMR testing of bacteria isolated from humans and farmed animals published by European Committee on Antimicrobial Susceptibility Testing (EUCAST) and CLSI were 35 °C ± 2 °C for 16–20 h. However, many bacteria isolated from aquatic animals, and capable of causing infections in those animals, grow poorly or do not grow under these conditions. As a consequence, the standard methods for AMR testing of bacteria from aquatic sources recommend incubation at 28 °C ± 2 °C for 24–28 h or 22 ± 2 °C for 24–48 h, depending on the species being examined [18]. An incubation temperature of 22 °C was chosen because this temperature represents the standard isolation temperature of fish pathogens in our laboratory and was maintained in the AMR testing. It is worth to note that the disk diffusion method is not the recommended method in order to determine AMR to colistin. A joint EUCAST and CLSI subcommittee issued recommendations confirming that broth microdilution is so far the only valid method and that disk diffusion does not work because of the poor diffusion of the large colistin molecule [19]. On the basis of this strong limit, the results for colistin must be evaluated with caution and certainly the sensitivity to colistin need to be investigated with more suitable methods in the future. For those antimicrobials where interpretative criteria are not available for *Aeromonas* spp., *Enterobacteriaceae* susceptibility criteria were applied [20]. *E. coli* ATCC 35218 and *E. coli* ATCC 25922 were included as control strains. Isolates that were resistant to at least one agent in three or more antimicrobial classes were considered MDR [21].

### Statistical analysis

The prevalence of AMR of motile *Aeromonas* strains was calculated and the binomial exact method was used to compute 95% confidence intervals (95% CI). For each isolate the number of AMR was determined and used for further analysis. The association between AMR and *Aeromonas* complex (*A. hydrophila*, *A. sobria, A. caviae*), source of the isolates (farmed or wild fish), or sampling year (2013, 2016) was evaluated by a chi-square test (χ^2^) or Fisher exact test when appropriate. Correlations between AMR profiles of *Aeromonas* spp. to tested antimicrobials were calculated by the Phi index. A logistic regression model was performed to calculate the probability for isolates to develop AMR due to the presence of each of the following variables: *Aeromonas* complex, strain source, year of sampling, and multidrug-resistant. For each test a P < 0.05 was considered statistically significant. All analyses were performed using R software (R version 3.3.1, R Development Core Team [https://www.R-project.org/]) [22].

## Results

### Phenotypic identification and source of isolates

All the isolates that were examined presented typical biochemical reactions and were allocated into one of three motile *Aeromonas* complexes. Of 95 *Aeromonas* isolates, 55.8% (53/95), 32.6% (31/95) and 11.6% (11/95) were identified as *A. hydrophila*, *A. sobria*, and *A. caviae*, respectively. *A. hydrophila* and *A. sobria* complexes were more prevalent within wild fish, with frequencies of 75.5% (40/53) and 80.6% (25/31), respectively, compared with *A. caviae* complex. Conversely, the latter was the most frequently isolated complex from farmed fish (63.6%, 7/11). Those differences were significant (χ^2^ = 8.47, P = 0.0078). No differences were found between the prevalences of the three *Aeromonas* complexes in 2013 and 2016 (χ^2^ = 2.49, P = 0.2871).

### Antimicrobial resistance

The AMR patterns of 95 *Aeromonas* isolates tested are shown in Table 1. All isolates were resistant to cloxacillin, spiramycin, and tilmicosin. High AMR frequencies (> 95%) were also observed for tylosin, penicillin and sulfadiazine. Conversely, resistances to danofloxacin, enrofloxacin, flumequine, ceftiofur, aminosidine, colistin, doxycycline, gentamicin, marbocyl, and florfenicol were observed at low levels (< 10%). None of the isolates were found to be resistant to cefquinome.

**Table 1:** AMR profiles of *Aeromonas* isolates

Antimicrobials	No. of resistant isolates (%)				Chi-square index (χ^2^)
	*A. hydrophila*	*A. sobria*	*A. caviae*	Total	
Cloxacillin	53 (100)	31 (100)	11 (100)	95 (100)	/
Spiramycin	53 (100)	31 (100)	11 (100)	95 (100)	/
Tilmicosin	53 (100)	31 (100)	11 (100)	95 (100)	/
Ampicillin*	53 (100)	31 (100)	10 (90.9)	94 (98.9)	7.72
Tylosin	53 (100)	30 (96.8)	11 (100)	94 (98.9)	2.09
Penicillin	52 (98.1)	30 (96.8)	11 (100)	93 (97.9)	0.44
Sulfadiazine*	53 (100)	28 (90.3)	11 (100)	92 (96.8)	6.40
Amoxicillin	52 (98.1)	30 (96.8)	10 (90.9)	92 (96.8)	1.55
Tiamulin**	48 (90.6)	22 (71.0)	11 (100)	81 (85.3)	8.13
Neomycin**	27 (50.9)	26 (83.9)	4 (36.4)	57 (60.0)	11.73
Erythromycin***	35 (66.0)	9 (29.0)	11 (100)	55 (57.9)	20.04
Cephaloridine***	38 (71.7)	3 (9.7)	10 (90.9)	51 (53.7)	37.19
AMC	27 (50.9)	11 (35.5)	5 (45.5)	43 (45.3)	1.89
Apramycin***	16 (30.2)	23 (74.2)	0	39 (41.1)	24.32
Tetracycline**	12 (22.6)	1 (3.2)	5 (45.5)	18 (8.9)	10.49
SXT**	14 (26.4)	0	2 (18.2)	16 (16.8)	9.76
Nalidixic acid*	12 (22.6)	0	3 (27.3)	15 (15.8)	8.77
Thiamphenicol*	11 (20.8)	0	2 (18.2)	13 (13.7)	7.35
Kanamycin	5 (9.4)	6 (19.4)	0	11 (11.6)	3.51
Danofloxacin	7 (13.2)	0	2 (18.2)	9 (9.5)	5.08
Enrofloxacin***	4 (7.5)	0	4 (36.4)	8 (8.4)	14.04
Flumequine	5 (9.4)	0	2 (18.2)	7 (7.4)	4.68
Ceftiofur***	1 (1.9)	1 (3.2)	4 (36.4)	6 (6.3)	19.04
Aminosidine*	1 (1.9)	5 (16.1)	0	6 (6.3)	7.54
Colistin	4 (7.5)	2 (6.5)	0	6 (6.3)	0.88
Doxycycline**	2 (3.8)	0	3 (27.3)	5 (5.3)	12.64
Gentamicin	3 (5.7)	0	0	3 (3.2)	2.45
Marbocyl	3 (5.7)	0	0	3 (3.2)	2.45
Florfenicol	2 (3.8)	0	1 (9.1)	3 (3.2)	2.34
Cefquinome	0	0	0	0	/

Significant differences between *Aeromonas* complexes were indicated by: * P < 0.05, ** P < 0.01, *** P < 0.001

Considering the antimicrobial classes, *Aeromonas* isolates showed the highest levels of AMR to macrolides (100%), penicillins (100%), sulfonamides (96.8%), and pleuromutilins (83.5%). Aminoglycoside resistance was also frequently detected (62.1%), while resistance to polymyxins and cephalosporins (third and fourth generations) was observed rarely (6.3%).

With regard to different *Aeromonas* complexes, variable profiles of AMR existed. Significant differences in AMR patterns were observed for 15 antimicrobials: aminosidine, ampicillin, apramycin, ceftiofur, cephaloridine, doxycycline, enrofloxacin, erythromycin, nalidixic acid, neomycin, sulfadiazine, SXT, tetracycline, thiamphenicol and tiamulin. In particular, strong associations were found between the complex *A. caviae* and resistance to apramycin (Phi = 0.51). In a multivariate analysis of data, complex-related variability resulted in statistical significance only for four agents: aminosidine, cephaloridine, ceftiofur, and neomycin. For the others, the effect of strain source or sampling year on AMR was stronger than that of belonging to a specific complex. The probability of observing AMR to aminosidine (OR 188.6, 95% CI 2.8–inf) and neomycin (OR 1.6, 95% CI 1.1–2.2) was higher in *A. sobria* than in the other two complexes. The probability of detecting resistance to cephaloridine was higher in *A. caviae* and *A. hydrophyla* than in an *A. sobria* complex (OR 30.2, 95% CI 7.5–121.9). Finally, the probability of finding AMR to ceftiofur was higher in *A. caviae* than the other two complexes (OR 17.1, 95% CI 1.7–178.1).

AMR patterns of *Aeromonas* spp. recovered from farmed and wild freshwater fish are displayed in Table 2. According to the source of isolates, significant differences in AMR frequencies were found for five agents: apramycin, doxycycline, nalidixic acid, sulfadiazine and tetracycline.

**Table 2:** AMR profiles of *Aeromonas* isolates recovered from farmed and wild freshwater fish

Antimicrobials	No. of resistant isolates (%)		Chi-square index (χ^2^)
	Farmed fish	Wild fish	
Cloxacillin	26 (100)	69 (100)	/
Spiramycin	26 (100)	69 (100)	/
Tilmicosin	26 (100)	69 (100)	/
Ampicillin	25 (96.2)	69 (100)	0.26
Tylosin	25 (96.2)	69 (100)	0.26
Penicillin	24 (92.3)	69 (100)	2.33
Sulfadiazine*	23 (88.5)	69 (100)	4.88
Amoxicillin	24 (92.3)	68 (98.6)	0.79
Tiamulin	19 (73.1)	62 (89.9)	3.00
Neomycin	15 (57.7)	42 (60.9)	0
Erythromycin	14 (53.8)	41 (59.4)	0.07
Cephaloridine	13 (50.0)	38 (55.1)	0.05
AMC	10 (38.5)	33 (47.8)	0.34
Apramycin*	6 (23.1)	33 (47.8)	3.81
Tetracycline**	10 (38.5)	8 (11.6)	7.21
SXT	3 (11.5)	13 (18.8)	0.29
Nalidixic acid*	8 (30.8)	7 (10.1)	4.59
Thiamphenicol	2 (7.7)	11 (15.9)	0.50
Kanamycin	1 (3.8)	10 (14.5)	1.18
Danofloxacin	5 (19.2)	4 (5.8)	2.56
Enrofloxacin	4 (15.4)	4 (5.8)	1.18
Flumequine	4 (15.4)	3 (4.3)	1.95
Ceftiofur	2 (7.7)	4 (5.8)	0
Aminosidine	0	6 (8.7)	1.17
Colistin	0	6 (8.7)	1.17
Doxycycline***	5 (19.2)	0	10.42
Gentamicin	2 (7.7)	1 (1.4)	0.79
Marbocyl	2 (7.7)	1 (1.4)	0.79
Florfenicol	2 (7.7)	1 (1.4)	0.79
Cefquinome	0	0	/

Significant differences between *Aeromonas* isolates of farmed and wild origin were indicated by: * P < 0.05, ** P < 0.01, *** P < 0.001

Nevertheless, according to multivariate analysis, the effects on the source of isolates in the AMR results were statistically significant only for three antimicrobials: nalidixic acid, apramycin, and tetracycline. *Aeromonas* spp. recovered from farmed fish showed a probability to be resistant to nalidixic acid that was about four times higher when compared to wild isolates (OR 3.9, 95% CI 1.3–12.3). Likewise, isolates of farmed origin were twice as likely to be resistant to tetracycline when compared to wild isolates (OR 2.1, 95% CI 1.4–3.2). Finally, isolates of wild origin exhibited a four times higher probability to be resistant to apramycin than farmed isolates (OR 4.2, 95% CI 1.4–12.6).

Statistical differences in AMR between *Aeromonas* spp. isolated in 2013 and 2016 were detected (Table 3). Overall, the isolates showed a significant increase in resistance to neomycin, AMC and apramycin while resistance to erythromycin, tetracycline, and thiamphenicol significantly decreased during the study period.

**Table 3:** AMR profiles of *Aeromonas* spp. isolated in 2013 and 2016

Antimicrobials	No. of resistant isolates (%)		Chi-square index (χ^2^)
	2013	2016	
Cloxacillin	31 (100)	64 (100)	/
Spiramycin	31 (100)	64 (100)	/
Tilmicosin	31 (100)	64 (100)	/
Ampicillin	30 (96.8)	64 (100)	0.14
Tylosin	31 (100)	63 (98.4)	0
Penicillin	31 (100)	62 (96.9)	0.05
Amoxicillin	29 (93.5)	63 (98.4)	0.43
Sulfadiazine	31 (100)	61 (95.3)	0.36
Tiamulin	27 (87.1)	54 (84.4)	0
Neomycin**	12 (38.7)	45 (70.3)	7.42
Erythromycin**	25 (80.6)	30 (46.9)	8.43
Cephaloridine*	23 (74.2)	28 (43.8)	6.61
AMC*	8 (25.8)	35 (54.7)	5.91
Apramycin**	6 (19.4)	33 (51.6)	7.67
Tetracycline*	10 (32.3)	8 (12.5)	4.1
SXT	8 (25.8)	8 (12.5)	1.78
Nalidixic acid	5 (16.1)	10 (15.6)	0
Thiamphenicol**	9 (29.0)	4 (6.3)	7.35
Kanamycin	2 (6.5)	9 (14.1)	0.56
Danofloxacin	1 (3.2)	8 (12.5)	1.15
Enrofloxacin	4 (12.9)	4 (6.3)	0.49
Flumequine	1 (3.2)	6 (9.4)	0.43
Aminosidine	1 (3.2)	5 (7.8)	0.17
Colistin	1 (3.2)	5 (7.8)	0.17
Ceftiofur	2 (6.5)	4 (6.3)	0
Doxycycline	2 (6.5)	3 (4.7)	0
Florfenicol	0	3 (4.7)	0.36
Gentamicin	1 (3.2)	2 (3.1)	0
Marbocyl	1 (3.2)	2 (3.1)	0
Cefquinome	0	0	/

Significant differences between *Aeromonas* spp. isolated in 2013 and 2016 were indicated by: * P < 0.05; ** P < 0.01; *** P < 0.001

No significant changes in AMR among farmed isolates were observed over the study period, with the exception of resistance to amoxicillin, which increased from 66.7% in 2013 to 100% in 2016 (Phi = 0.53) (data not shown). In contrast, a significant increase in resistance to neomycin, AMC, and apramycin among *Aeromonas* spp. from wild fish was observed during the study period while resistance to erythromycin, tetracycline and thiamphenicol significantly decreased (data not shown).

### Multidrug resistance

**Fig. 1:**
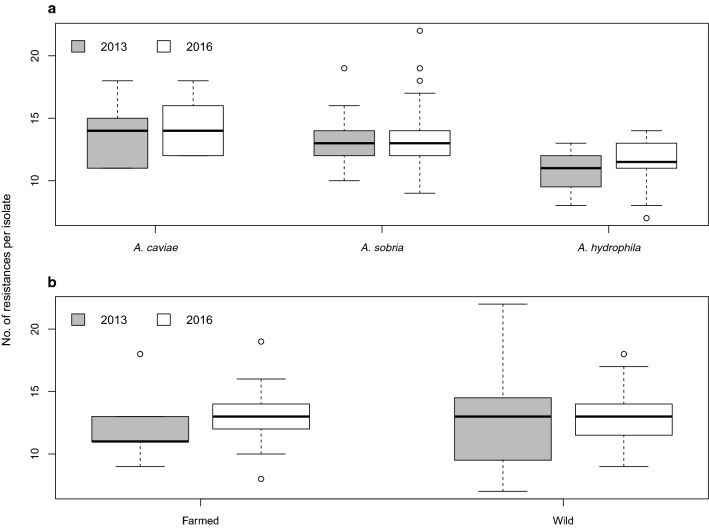
Distribution of AMR per each *Aeromonas* isolate by **a** sampling year and complex and **b** by sampling year and source of isolates

Regardless of *Aeromonas* complex and the source of *Aeromonas* spp., all isolates were MDR. Moreover, each isolate showed a pattern of resistance to at least seven antimicrobials. The mean resistance value to tested antimicrobials was 12.7 (± 2.7 ds; range 7–2; median 13). The minimum number of resistances (seven antimicrobials) was observed in two isolates, both belonging to the *A. sobria* complex. The maximum number of resistances (22 antimicrobials) was found in one isolate, belonging to the *A. hydrophila* complex. Figure 1 shows the distribution of AMR per each *Aeromonas* isolate by sampling year, complex and source. Our results indicated that there was a tendency towards a higher number of resistances among *A. caviae* isolates compared to other two complexes, both in 2013 and 2016. Considering the source of *Aeromonas* spp., an increase in resistance rates among isolates from farmed fish was observed during the study period, while isolates of wild origin tended to exhibit the same pattern over the time.

## Discussion

In this study, fish isolates of motile *Aeromonas* spp. from northern Italy displayed high levels of resistance to various antimicrobials agents. Regardless of *Aeromonas* complex, resistance to penicillins, macrolides, pleuromutilins and sulfonamides was particularly widespread and it was detected in all or almost all the isolates. The high resistance to penicillins observed here is in agreement with the intrinsic resistance of *Aeromonas* species to beta-lactams widely reported in the literature; in fact, *Aeromonas* bacteria are known to be intrinsically resistant to aminopenicillins and first generation cephalosporins.

This is due to the production of multiple, inducible, chromosomally encoded beta-lactamases [23, 24]. According to the source of isolates, very few differences in AMR rates were observed between aeromonads recovered from farmed and wild fish. Among antimicrobials registered for use in Italian aquaculture and tested here (i.e., amoxicillin, flumequine, SXT, tetracycline), only tetracycline showed higher AMR in isolates from farmed fish than those from wild ones. Moreover, except for amoxicillin, no significant trend of increased resistance among isolates of farmed origin was observed during the study period. These results suggest that the antimicrobial use in aquaculture should not be the only contributing factor considered when evaluating the AMR of *Aeromonas* species from fish.

Excluding the intrinsic AMR to beta-lactams, the high AMR frequencies observed here could be explained by acquired mechanisms involving the transfer of antibiotic resistance genes (ARGs) from other aquatic bacteria [25]. In this research, all sampling sites (fish farms and natural water bodies) are located near urban centres and livestock-producing areas. Thus, it is possible that some of the AMR resulted from the release of AMR bacteria and drugs residues from urban and animal wastewater being discharged into the aquatic ecosystems and their variations could reflect local antimicrobial usage. Once these bacteria are in the water environment, the exchange of ARGs among aquatic microorganisms through mobile genetic elements (MGEs), such as plasmids, is readily facilitated [26]. This outcome can result in a higher frequency of MDR isolates. There is concern that ARGs are widespread not only among clinical pathogens but also in environmental bacteria, as a consequence of the wide usage of antimicrobials in clinical practice, animal husbandry and agriculture [26–28].

With regard to *Aeromonas* complexes, our data revealed the presence of significant differences in the AMR, suggesting that phenotypic identification of motile aeromonads at complex level may be useful in clinical practice for the selection of the first line of antimicrobial to be administered in infections caused by these bacteria. Moreover, phenotypic methods have the advantage of being particularly suitable for use in diagnostic routine activity because it uses standard biochemical tests [14].

However, it is worth noting that AMR profiles of isolates tested showed a considerable high variability, which is likely due to the relatively small number of isolates included in the research. This can potentially affect the reliability of the results, which may not reflect the real resistance patterns of *Aeromonas* populations in freshwater habitats. Thus, data on AMR of these microorganisms should be further investigated through epidemiological studies based on a larger sample size.

The frequent occurrence of MDR aeromonads observed in this study is of concern considering the ubiquitous nature and the potential role as reservoir of ARGs of *Aeromonas* spp. [27, 29]. Motile species also have a zoonotic potential, causing gastroenteritis, skin and soft tissue infection, and bacteraemia in immunocompromised patients [1]. In this regard, the detection of various degrees of resistance even to drugs listed in the highest priority, critically important antimicrobial classes in human medicine (macrolides, quinolones, fluoroquinolones, polymyxins, third and fourth generations of cephalosporins) [30] is of particular concern. Among these classes, quinolones are even considered the antimicrobials of choice to treat human *Aeromonas* infections [31].

## Conclusions

Our findings contribute to highlight the role of freshwater fish as reservoir of MDR motile *Aeromonas* spp., which can represent a potential hazard to consumers via food-borne infections and a risk factor for wound infections after handling contaminated fish (e. g. in the case of aquaculture workers, fish handlers, or aquarium owners).

The broad distribution of these bacteria in different habitats remains a relevant public health issue. Therefore, there should be a continuous and regular effort to monitor the dissemination of resistant aeromonads in aquatic ecosystems on a global scale, evaluating different environmental sources.

## Data Availability

The datasets used and/or analysed during the current study are available from the senior (last) author on reasonable request.
